# Study of the Fatigue Crack Growth in Long-Term Operated Mild Steel under Mixed-Mode (I + II, I + III) Loading Conditions

**DOI:** 10.3390/ma13010160

**Published:** 2020-01-01

**Authors:** Grzegorz Lesiuk, Michał Smolnicki, Dariusz Rozumek, Halyna Krechkovska, Oleksandra Student, José Correia, Rafał Mech, Abílio De Jesus

**Affiliations:** 1 Department of Mechanics, Material Science and Engineering, Wroclaw University of Science and Technology, Smoluchowskiego 25 Wrocław, PL-50372 Wrocław, Poland; michal.smolnicki@pwr.edu.pl (M.S.); rafal.mech@pwr.edu.pl (R.M.); 2 Department of Mechanics and Machine Design, Opole University of Technology, Mikołajczyka 5, PL-45271 Opole, Poland; d.rozumek@po.edu.pl; 3 Karpenko Physico-Mechanical Institute of the National Academy of Sciences of Ukraine, Naukova 5, 79060 Lviv, Ukraine; galyna@ipm.lviv.ua (H.K.); student@ipm.lviv.ua (O.S.); 4 INEGI, Faculty of Engineering, University of Porto, Rua Dr. Roberto Frias, Campus FEUP, 4200-465 Porto, Portugal; ajesus@fe.up.pt

**Keywords:** mixed-mode fracture, fatigue crack growth, crack growth rate, finite element analysis, crack paths, crack closure, fractography

## Abstract

The paper presents an analysis of mixed-mode fatigue crack growth in bridge steel after 100-years operating time. Experiments were carried out under mode I + II configuration on Compact Tension Shear (CTS) specimens and mode I + III on rectangular specimens with lateral stress concentrator under bending and torsion loading type. Due to the lack of accurate Stress Intensity Factor (SIF) solutions, the crack path was modelled with the finite element method according to its experimental observation. As a result, the Kinetic Fatigue Fracture Diagrams (KFFD) were constructed. Due to the change in the tendency of higher fatigue crack growth rates from K_I_ towards K_III_ dominance for the samples subjected to bending and torsion, it was decided to analyze this phenomenon in detail using electron-scanning microscopy. The fractographic analysis was carried out for specimens subjected to I + III crack loading mode. The mechanism of crack growth in old bridge steel at complex loads was determined and analyzed.

## 1. Introduction

Fatigue fracture is one of the most frequent failure reasons for bridge structures. It is worth noting that the research guidelines [1] for old bridge structures are conservative as regard fatigue, namely as concerns the sub-critical period of fatigue crack growth. Of course, this is motivated by the absence of reliable data from old bridge materials, especially original data. The maintenance of historic bridges is still an important issue, especially justified by the fact that every year, the number of structures with more than 100 years of service life grows. It is generally assumed that until the end of the 19th century, the construction material was the puddle iron. Many papers have been devoted to the puddle iron [2,3,4,5,6] in the field of material characterization, including fatigue testing. While state of the art and test procedures are clear for uniaxial loads, despite the diversity of results, there is little work on the multi-axial fatigue of this type of material. Papers [7,8,9] of the authors’ team devoted to the mixed-mode (I + II) fatigue fracture of puddle iron, show significant discrepancies in the expected fatigue-fracture behavior of puddle iron compared with existing mixed-mode criteria. On the other hand, realistic assessment of the structural integrity using modern tools such as the finite element method [10,11,12] requires the substitution of growth models for uniaxial states with models for mixed fracture growth modes. The development of metallurgy at the beginning of the 20th century has enabled the manufacture of homogenous materials with parameters similar to modern low carbon structural steels. However, many papers [13,14,15] reported the deterioration of mechanical properties caused by the phenomenon of microstructural degradation. In the case of old bridge steels, the same material properties as for modern steels are often assumed. Similarly to modern structural steels, the fatigue crack propagation behavior studies have been limited to uniaxial state analyzes. This paper is intended to contribute to fill the existing gap in literature analyzing the fatigue crack growth process for typical fatigue crack load schemes, i.e., tension and shear (I + II), occurring at the riveted crack initiation points [16], and bending and torsion (I + III), occurring at the bridge structural members [17]. Due to the lack of available experimental data for objects erected at the beginning of the 20th century, it was decided to focus the research on material samples from a structural part extracted from a repaired existing steel bridge (1899–1902) located in Poland [18].

## 2. Materials and Methods

### 2.1. Material Data

**Figure 1:**
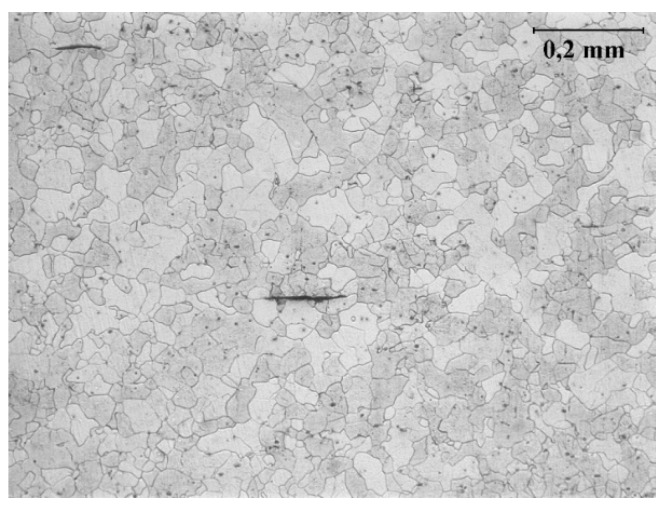
The microstructure of the tested long-term operated mild steel.

**Figure 2:**
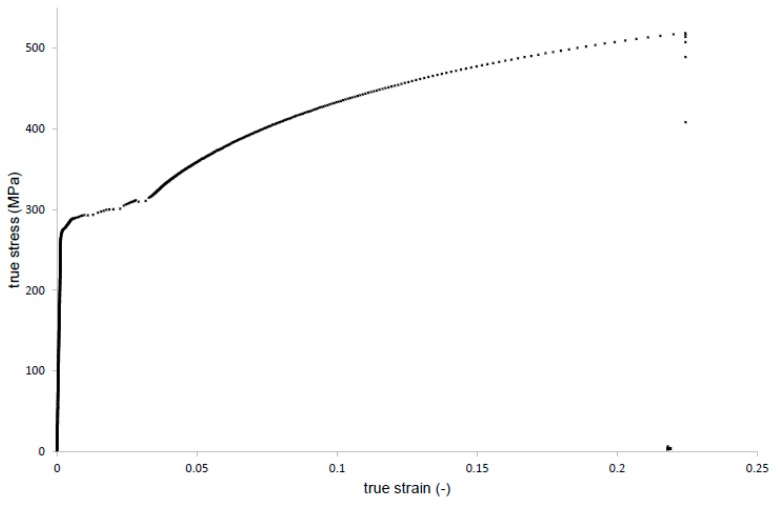
Representative room temperature tensile true stress-true strain curve obtained from the uniaxial tensile test for the old mild steel.

For mixed-mode fatigue crack growth studies, old mild steel extracted from the old railway bridge is considered. The mentioned bridge is part of railway line 143 (Kalety–Wrocław Mikołajów) and is located in Kluczbork, Poland. The chemical composition analysis was carried out in order to identify the tested steel. Chemical composition of this steel, as well as reference values of typical puddle iron and old mild steel are presented in Table 1. Presented data suggested that the steel from Kluczbork Bridge belongs to an old mild steel group. Before mechanical testing, the metallographic study was performed for the analyzed material. The microstructure of the tested steel is shown in Figure 1. A ferrite grain structure with non-metallic inclusions is observed, corresponding well with carbon content. Static tensile testing was also performed and reported in [18]. A representative true stress–strain curve is shown in Figure 2, from which is reported the following mechanical properties [18]: Young modulus, E=212 GPa; ultimate tensile strength,$\;\sigma_{u}=416\;\mathrm{MPa}$; yield tensile strength, Re=304 MPa.

### 2.2. Mixed-Mode Fatigue-Fracture Characterization—Experimental Details

**Figure 3:**
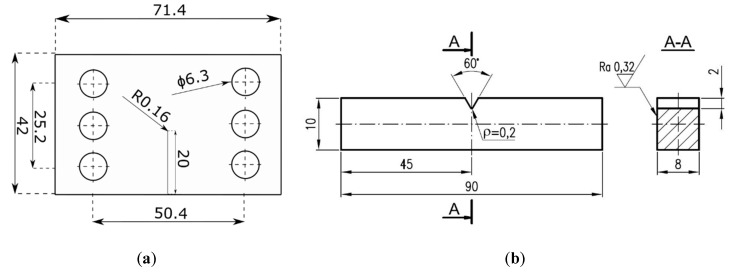
Geometry and dimensions (in mm) of specimens used in both the experimental and numerical campaign: (**a**) CTS (Compact Tension Shear) specimen (thickness—8 mm); (**b**) specimen subjected for mode I + III testing.

In order to assess the fatigue-fracture behavior under mixed-mode loading conditions (I + II, I + III), two kinds of experiments were performed in order to determine the fatigue crack propagation rates. For the experimental campaign, two types of specimen were designed (Figure 3). In Figure 3a, the main dimensions of Compact Tension Shear (CTS) specimens according to the original concept of Richard [19] are shown. For mode I + III, the prismatic specimen with the lateral notch was designed and prepared, see Figure 3b.

#### 2.2.1. Mode I + II Test

CTS specimens used in this study presented an initial notch with dimensions: Length, l=20 mm and root radius, ρ=0.16 mm. Detailed geometry of the used specimen is presented in Figure 3a. These notches were cut using electro-discharged machining (EDM).

**Figure 4:**
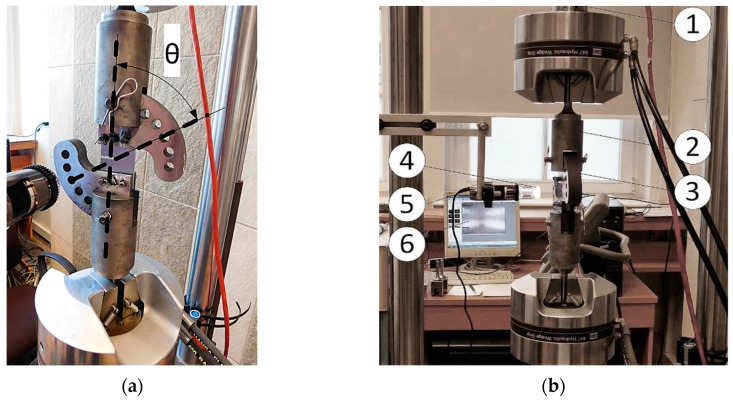
Experimental mixed-mode I + II loading test: (**a**) Definition of load angle, θ; (**b**) measurement stand: 1—load cell, 2—gripping system, 3—CTS clevis, 4 – light source, 5—digital microscope, 6—imaging system.

The main advantage of the CTS specimen is the fact that the mixed-mode loading condition can be performed using a uniaxial hydraulic pulsator. The experimental setup is presented in Figure 4. An unique clevis and gripping system are necessary for testing the CTS specimen. Due to changing the θ angle (defined in Figure 4a), it is possible to generate pure mode I experiment (θ = 0°) as well as “pure shear” state (θ = 90°). Between θ = 0° and θ = 90° mixed-mode loading conditions are generated. All specimens were pre-cracked under pure mode I condition and preserving the linear elastic fracture mechanics conditions. After pre-cracking, the proper mixed-mode experiments were performed for load angle θ = 30° and θ = 45°, respectively. Experimental setup (see Figure 4b) consists of a 100 kN load cell (1), clevis (2), CTS specimen holder (3), light source with polarized filters (4), DinoLite Microscope (5) as well as integrated measurement system operated by computer with MTS FlexTest controller software (6). Tests were conducted with a frequency, f=10 Hz, constant force amplitude, F=10 kN, and load ratio, R=0.1. During the experiment, an additional imaging system was involved in order to periodically capture crack tip point images.

#### 2.2.2. Mode I + III Test

**Figure 5:**
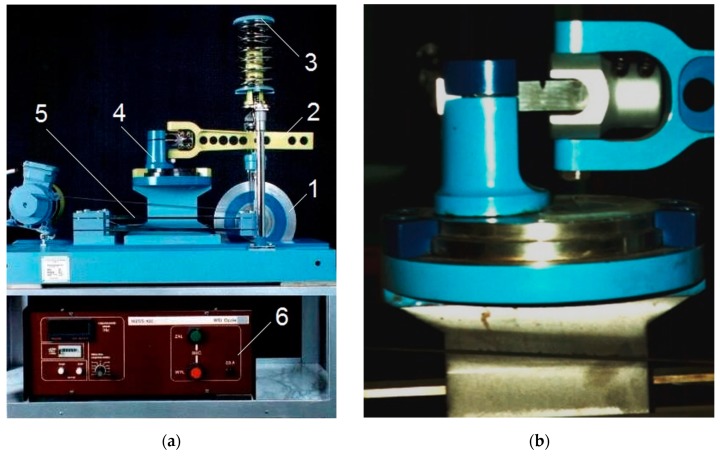
Fatigue test stand MZGS-100: (**a**) Overview; (**b**) specimen during the test.

The specimens subjected to mixed mode (I + III) condition showed an external, unilateral notch, which was $a_{0}=2\;\mathrm{mm}$ deep and its root radius was, *ρ* = 0.2 mm. The notches in specimens were cut with a cutter, and their surfaces were polished with progressively finer emery papers. The tests combining bending with torsion (mode I + III) were performed on the fatigue test stand MZGS–100 [20,21,22], enabling the realization of cyclically variable and static (mean) loading. The fatigue test stands MZGS–100 (Figure 5, Opole University of Technology, Opole, Poland) consists of power, control, and loading units. Cyclic loading is obtained by vertical movements of the lever (2) motion in the vertical plane, generated by the inertial force of the rotating disk (1) mounted on flat springs (5). A spring-loaded actuator (3) is fixed to the fatigue testing machine base. The tests were conducted under controlled force (the amplitude of bending moment was controlled) with the frequency of 28.4 Hz. The tests were performed at a constant moment amplitude $M_{a}=17.19\;N\cdot m$ and stress ratio, R=0. The specimen during a test is shown in Figure 5b. The crack growth was observed by an optical method on specimen lateral surfaces with a magnification of 25 times, by recording the number of load cycles through the control box (6). Proportional bending with torsion, for α = 30° and 45°, was obtained after proper head rotation (4).

### 2.3. Stress Intensity Factors Calculations

In order to evaluate experimental results, stress intensity factors are needed (to calculate values of $\Delta K_{I,II,III}$). In literature, some formulas can be found for CTS specimen cases (Equations (1) and (2)) [19,23]. Nevertheless, these formulas are only valid for initial conditions, i.e., when the crack tip is in the symmetry plane of specimen:

(1) $$K_{I}=\frac{F\cdot \sqrt{\pi a_{0}}\cdot \cos\theta }{Wt\left(1-\frac{a_{0}}{W}\right)}\sqrt{\frac{0.26+2.65\left(\frac{a_{0}}{W-a_{0}}\right)}{1+0.55\left(\frac{a_{0}}{W-a_{0}}\right)-0.08{\left(\frac{a_{0}}{W-a_{0}}\right)}^{2}}}$$

(2) $$K_{II}=\frac{F\cdot \sqrt{\pi a_{0}}\cdot \sin\theta }{Wt\left(1-\frac{a_{0}}{W}\right)}\sqrt{\frac{-0.23+1.4\left(\frac{a_{0}}{W-a}\right)}{1-0.67\left(\frac{a_{0}}{W-a_{0}}\right)-2.08{\left(\frac{a_{0}}{W-a_{0}}\right)}^{2}}}$$

where: *F*—applied force, *a*_0_—initial crack length (notch + precrack), *θ*—loading angle, *W*—specimen width.

Analogous formulas for specimens prepared for mode I and III tests are presented in Equations (3) and (4) [21]:

(3) $$K_{I}=Y_{I}\sigma cos^{2}\alpha \sqrt{\pi \left(a_{0}+a\right)}$$

(4) $$K_{III}=Y_{III}\sigma cos\alpha sin\alpha \sqrt{\pi \left(a_{0}+a\right)}$$

In Equations (3) and (4), σ represents stress level, α the loading angle, and $Y_{I}$ and $Y_{III}$ are dimensionless geometric factors defined as [24,25]:

(5) $$Y_{I}=\frac{5}{\sqrt{20-13\left(\frac{a_{0}+a}{w}\right)-7{\left(\frac{a_{0}+a}{w}\right)}^{2}}}$$

(6) $$Y_{III}=\sqrt{\frac{2w}{a_{0}+a}\tan\frac{\pi \left(a_{0}+a\right)}{2w}}$$

**Figure 6:**
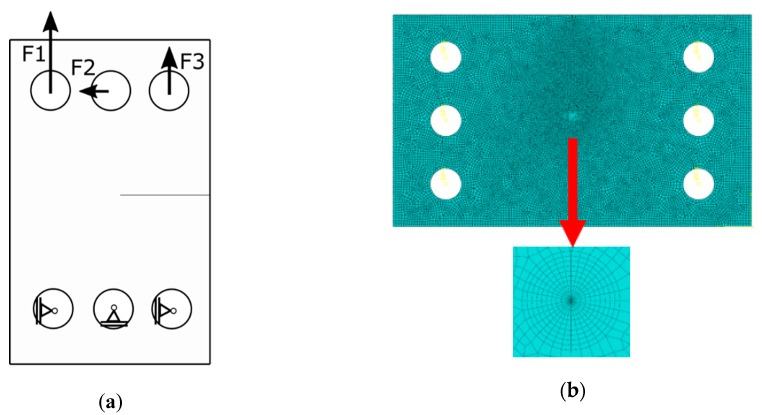
CTS specimen numerical analysis: (**a**) Boundary conditions; (**b**) finite element mesh with detail overview near the crack tip.

**Figure 7:**
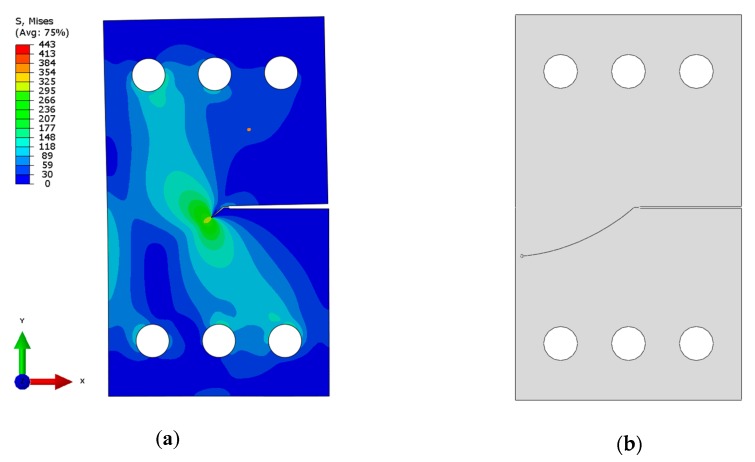
(**a**) Huber–Mises stress distribution in CTS specimen (load angle θ = 45°) with 3 mm long mixed-mode fatigue crack length; (**b**) predicted (posterior—based on the real crack path) crack trajectory in the discrete model used for stress intensity factors calculation.

**Figure 8:**
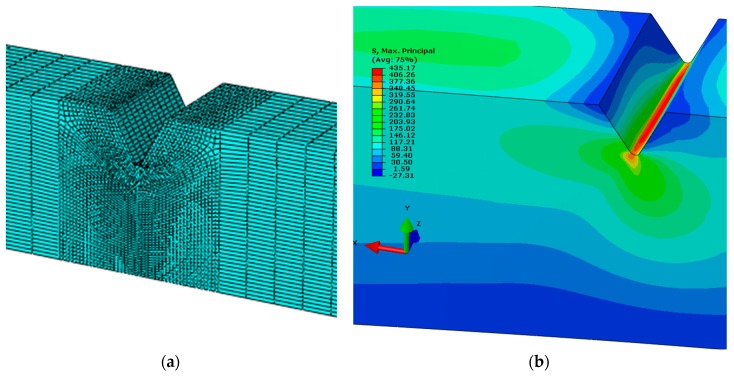
Rectangular specimen subjected to mode I + III testing: (**a**) Notch mesh density; (**b**) principal stress distribution at the initial stage of the crack growth for load angle, *α* = 30°.

Due to the limitations of previous formulae, the finite element method (FEM) was applied to support the experimental investigations. Abaqus FEM code (version 6.13-2) was used in all cases. The two-dimensional specimen was modelled using a geometry like the one shown in Figure 6, Figure 7 and Figure 8. For the CTS specimen, boundary conditions used in the analysis are presented in Figure 6a. Force components were defined to show a resultant force in the desired direction and a null resultant moment. Forces satisfying these conditions were calculated as presented in Equations (7)–(9). Pins were modelled as kinematic couplings, but rotation about the z-axis was allowed to prevent over stiffness. Discretization was made mostly using CPS4R elements (4-noded bilinear plane stress quadrilateral elements with reduced integration and hourglass control). Near the crack tip, triangular elements were used to ensure that contours will be of circular shape. The Contour Integral Method requires that all elements are of the same type, so triangular elements were automatically converted from quadrilaterals with one side collapsed. The finite element mesh is presented in Figure 6b. Stress intensity factors were calculated using the Contour Integral Method. To handle singularity problem, nodes at the crack tip were translated by 0.25 times of the element length. The whole process of determining stress intensity factors during crack growth consisted of simulation for every observed crack growth increment. During the simulation, the re-meshing approach has been adopted. However, it is worthwhile to underline that the presented approach is sensitive to a length scale. Therefore, the authors selected this crack growth increment that allows predicting results in the same manner as in the experimental campaign. For every simulation, the direction of fatigue crack growth and crack growth increment corresponds with the real crack paths registered during the experiments.

A similar approach was successfully implemented in previous authors’ papers devoted to mixed mode fatigue crack growth in 19th century puddle iron [7,8]. For modern constructional steels [21], the MTS (Maximum Tangential Stress) criterion works well enough, and it allows for the prediction of the fatigue crack path in the numerical environment, but in case of the long-term operated metallic material, it can lead to errors [7,8]. On the other hand, for the long-term operated materials, it is more reasonable to implement real crack paths (and crack growth increments) from the experiments. This approach was selected because the criterion used in Abaqus (version 6.13-2) allows for the prediction of the crack growth direction using conventional SED (Strain Energy Density) or MTS criteria. In the presented case, the MTS criterion does not predict well enough fatigue crack growth direction—see results in Section 3.1. This procedure was automated using an Abaqus script (version 6.13-2) written in Python (version 2.6.2). SIFs obtained in that way are in reasonable conformity with analytical formulas (Equations (1) and (2)) for the cases where these formulas are valid. Figure 7 illustrates the Huber–Mises stress distribution in a CTS specimen loaded at a load angle, θ = 45°. The same figure also represents a geometric model defined for a real crack measurement. This geometric model is afterwards used to compute the the SIF using the described numerical model.

(7) $$F_{1}=F\cdot \left(0.5\cos\alpha +\frac{c}{b}\sin\alpha \right)$$

(8) $$F_{2}=F\cdot \sin\alpha$$

(9) $$F_{3}=F\cdot \left(0.5\cos\alpha -\frac{c}{b}\sin\alpha \right)$$

Numerical simulations using the Finite Element Method for the mixed-mode I + III condition were also performed based on a solid model. The discrete model of the specimen is shown in Figure 8a. The mesh is coarse in the gripping part and more refined in the notch volume. The boundary conditions are established by fixing one side of the specimen and applying concentrated load to imitate bending and torsion on the other side into a reference point. This reference point was then connected to the specimen by a kinematic coupling.

## 3. Results

**Figure 9:**
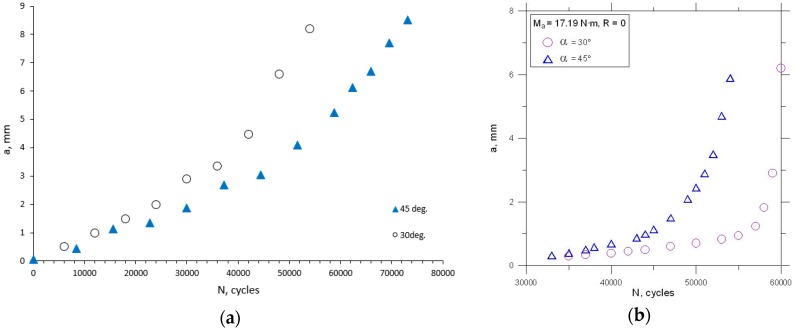
Fatigue crack growth curves for: (**a**) Mode I and II; (**b**) mode I and III.

**Figure 10:**
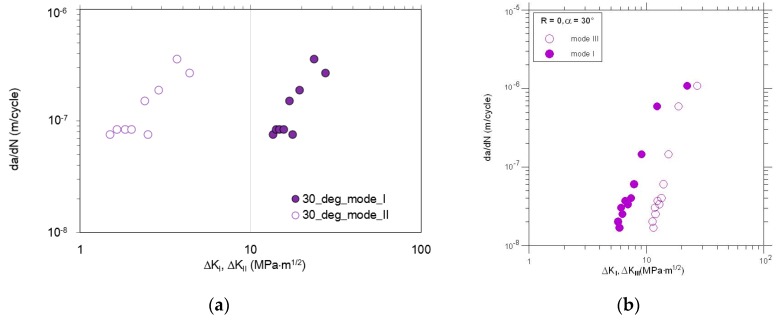
Comparison of the fatigue crack growth rates vs. ΔK vs. for α=θ=30° : (**a**) Mode I and II; (**b**) mode I and III.

**Figure 11:**
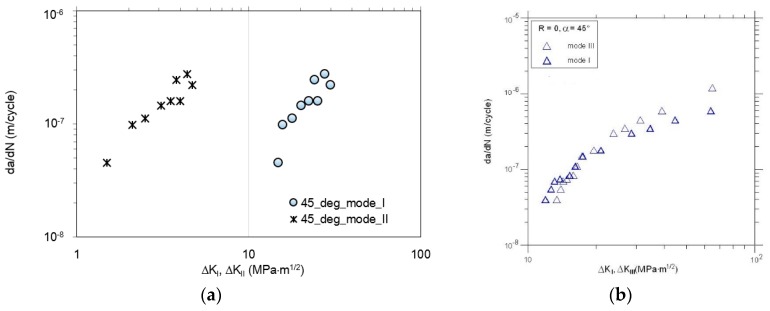
Comparison of the fatigue crack growth rates vs. ΔK for α=θ=45° : (**a**) Mode I and II; (**b**) mode I and III.

In general, the nonlinear crack path can be observed during experiments, and its plastic deformation in the last part of the crack path. Due to the plane constraint of the specimen and linear elastic fracture mechanics (LEFM) limitations, not all crack tip points from real crack paths were considered in all calculations. This posterior crack path was generated step-by-step via python script in Abaqus Simulia (version 6.13-2) environment in order to provide good conformity with a real crack growth trajectory. From the experiment, the a-N curves were collected, see Figure 9. Experimentally determined fatigue crack growth curves were analyzed for θ = *a* = 30°, and θ = *a* = 45°. Then, kinetic fatigue-fracture diagrams (KFFD) including crack growth rates, $\frac{da}{dN}$ were constructed as a function of $\Delta K_{i}$ for different loading modes i. The KFFDs are presented in Figure 10 and Figure 11.

It is worth to note that for increasing load angle (for all combinations of modes I + II and I + III) the higher fatigue lifetime of the specimen is observed. As it is also noticeable, for each case of mixed-mode I + II testing, the fatigue crack growth rates are higher for mode II in comparison with mode I. According to the KFFDs, the same slope of both curves seems to confirm a similar mechanism of fatigue crack growth for lower and higher mode mixity level. On the other hand, in the case of the combination mode I + III, it is noticeable that fatigue crack growth rate is higher for mode I in case of loading angle, *a* = 30°. However, increasing the mode mixity level and shear stress state for *a* = 45° seems to change this tendency, for a relatively high level of *K* > 18 MPa⋅m^0.5^. This phenomenon should be reflected in the topography of the fatigue-fracture surfaces.

### 3.1. Fatigue Crack Paths Study and Sem Analysis of Fracture Surfaces for Mixed-Mode I + III Loading

According to reference [26], the most widely used crack branching criterion is the Maximum Tangential Stress (MTS) criterion, where the initial angle of crack growth satisfies the following Equation (10):

(10) $$K_{I}\sin\theta +K_{II}(3\cos\theta -1)=0$$

and finally, allow determining the initial angle *ψ* of mixed-mode fatigue cracking:

(11) $$\tan{\left(\frac{\psi }{2}\right)}_{1,2}=\frac{K_{I}}{4K_{II}}\pm \frac{1}{4}\sqrt{{\left(K_{I}/K_{II}\right)}^{2}+8}$$

**Figure 12:**
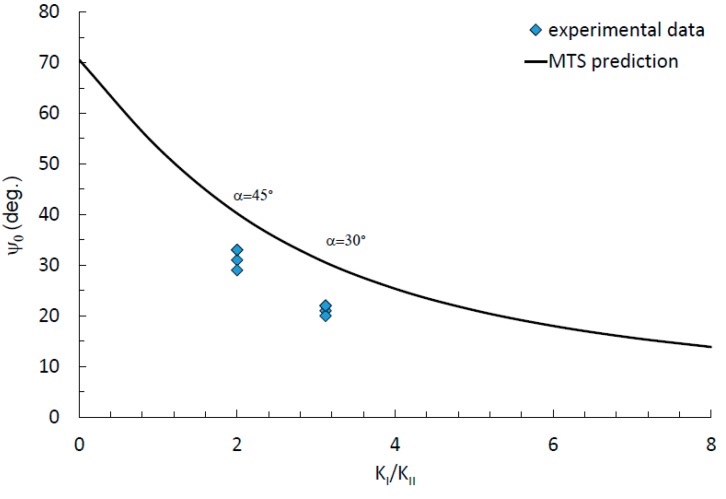
Crack initiation angles**,** according to MTS (Maximum Tangential Stress) criterion and experimental data for mode I + II.

Figure 12 shows the crack propagation angles measured after experiments in cracked CTS specimens along with predictions based on MTS criterion.

As it is noticeable, the initial fatigue crack angles do not follow the MTS criterion, which suggests further modification of the crack initiation angles prediction models. This situation can be associated with material damage accumulation after 100 years of operation. In the case of long-term operated steels, this phenomenon should be further investigated.

**Figure 13:**
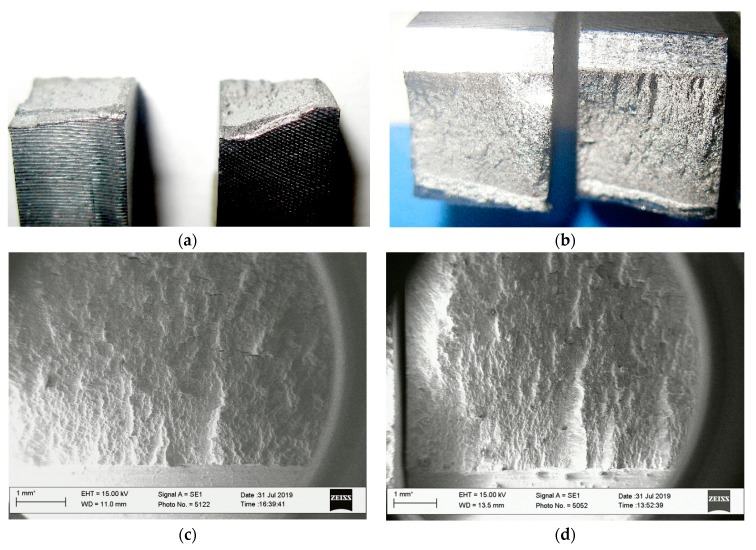
Profiles of the fracture surfaces of mode I-III test specimens: (**a**) Overview; (**b**–**d**) fracture features on the macro-level of the specimens.

On the other hand, fatigue tests at mixed I-III loading show that the smaller the amplitude of mode III loading (through deviation of the movable capture of specimens at the angles of 30 and 45 degrees), the higher the fatigue life of the specimens (6 × 10^4^ and 5.5 × 10^4^, respectively). Investigations of the topography of the fatigue-fracture surfaces of specimens show a significant effect of component III already at the macro level. During fatigue tests at mode I, fracture surfaces are usually formed normally oriented with respect to the direction of the acting stresses. Whereas, under the combined action of bending and torsion, the macro fracture surfaces of the specimens deviated from this orientation when approaching to their side surfaces. Moreover, the higher amplitude of the loading component K_III_, the greater the angle of deviation of the fracture surface was observed (Figure 13a). Also, this higher amplitude caused the more enormous heights of the ridges in the main direction of crack growth formed on the fracture surfaces (Figure 13b–d). These ridges correspond to the interface of local parts of the fracture surfaces with different orientations relative to the normal orientation in the center of the section of the specimens along with their thickness. Such a stepwise transition from one local part of the fracture surface to another ultimately ensured its deviation at the macro level of the regarding observed near side surfaces of specimens.

The zones of the initial stage of the fatigue crack growth, its accelerated growth, when the angle of inclination of the fracture surfaces became more noticeable, and spontaneous growth zones were observed on the macro fracture surfaces of the specimens (Figure 13a). The fatigue crack growth features at the micro-level were analyzed at the center parts of the fracture surfaces of the specimens by their thickness and near their side surfaces.

**Figure 14:**
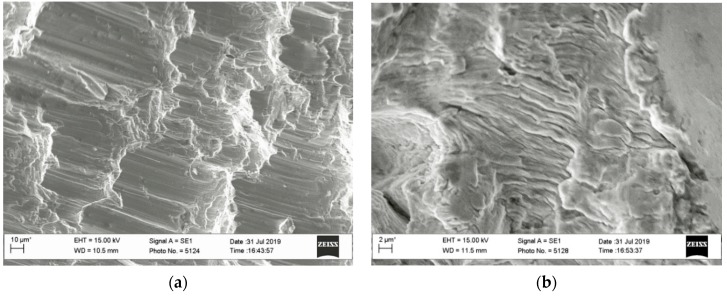
Typical illustrations of the fatigue-fracture surfaces of a specimen tested at a minimum amplitude of *K_III_* type (30 degrees): (**a**) Traces of contact between the conjugated surfaces in the load cycle; (**b**) fatigue striations at the final stage within starting zone of the crack growth (at the crack length near 2 mm) in the middle of the specimen thickness.

A common feature of all fracture surfaces was their damaging due to the contact of the conjugated fracture surfaces in the loading cycle (Figure 14a), due to the crack closure effect. The fracture surface obtained at the minimum loading amplitude according to the mode III loading type (at 30 degrees) was the most damaging due to the crack closure effect. The remains of an undamaged fatigue relief were found on this fracture surface only along the centerline of the specimen thickness. This is due to the smallest height of the ridges at the interfaces of adjacent local parts on the fracture surfaces due to the gradual reorientation of their inclination to the fracture surface. It is clear that they were completely damaged by the friction of surfaces during the specimen test. Therefore, the residues of the typical fatigue relief (undistorted by the friction of surfaces during this specimen test) could be only observed in the deepenings at the base of the ridges on this fracture surface. In this case, the small fragments with typical relief of fatigue striations perpendicular to the main direction of crack growth were observed (Figure 14b).

**Figure 15:**
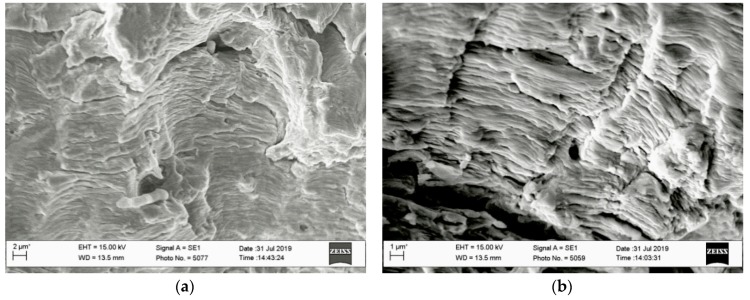

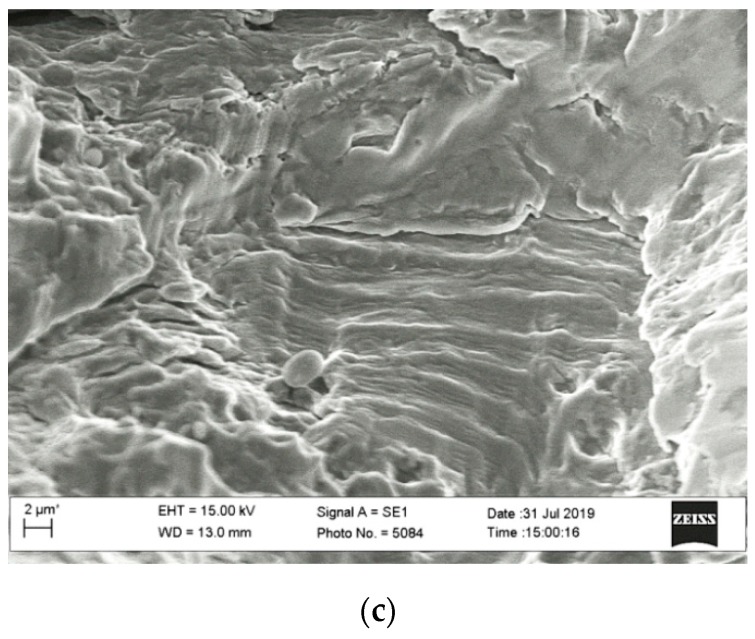
Fractograms in the center of the specimen thickness of the fracture surface: (**a**) Within the starting fracture zones; (**b**–**c**) within the zones of accelerated crack growth of the specimens, *K_I_*–*K_III_* with load angle *a* = 45°.

Fracture surfaces of the specimens tested at higher loading amplitudes of *K_III_* type (at 45 degrees) were more suitable for fractographic studies. The higher height of the ridges on the fracture surfaces connecting with their reorientation under the influence of the *K_III_* type loading component enables for the preservation of a higher number of fracture areas, with typical fatigue relief observed against the background of artefacts formed during contact of the mating surfaces in each loading cycle. At both loading amplitudes under the *K_III_* type, the typical fatigue features were observed on the fracture surfaces. In particular, among such features were the festoons elongated in the macro direction of crack growth. Within each of them, the same direction of local crack growth was observed, and fatigue striations oriented across them covered their surfaces (Figure 15). The influence of the load, according to *K_III_* type, was manifested in the following. At the loading of specimens according to K_I_ type, the flat festoons delimiting the areas with locally unidirectional crack growth are usually observed [27]. However, under the influence of *K_III_* type, loading the surfaces of each from the festoons revealed the fracture surfaces of specimens were curved. This was especially noticeable in the transitions between adjacent festoons. Obviously, during the testing of specimens according to the *K_I_*–*K_III_* type, the festoons were not merged by cleavage way (as it takes place after testing at the *K_I_* type loading), but due to the plastic stretching of the bridges between adjacent festoons with the formation of curvilinear ridges between them. The role of the loading amplitude according to scheme *K_III_* is almost levelled at the stage of accelerated crack growth with the expected increase of the spaces between fatigue striations.

**Figure 16:**
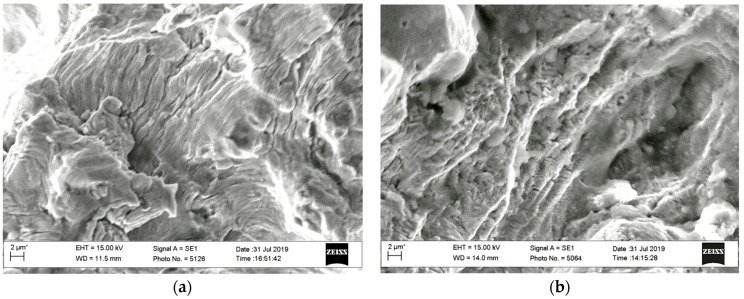
Fractograms of specimens obtained at a distance between 1 and 3 mm from their side-surfaces at the final stage of the starting fracture zones of specimens loaded at *K_I_*–*K_III_* type: (**a**) Up to 30 degrees by *K_III_*; (**b**) up to 45 degrees by *K_III_*.

The most interesting difference between the analyzed fracture surfaces from the observed specimens loading under *K_I_* type consists of the following. With the specimens tested at the *K_I_*–*K_III_* loading type, the direction of crack propagation is changed with its growth into the deep of the cross-section of specimens. In the middle of their thickness, the traditional mutually perpendicular orientation of the fatigue striations regarding the main direction of crack growth is observed. Near the end of the starting fatigue zone, more and more areas were recorded on the fracture surfaces with other orientations (Figure 16a,b). The orientation of the fatigue striations gradually changed, and at approaching the side surfaces of the specimen, it could even become parallel to the main direction of crack growth. In other words, the crack propagation from the middle part of the specimen along their thickness in the directions to the side surfaces has begun.

**Figure 17:**
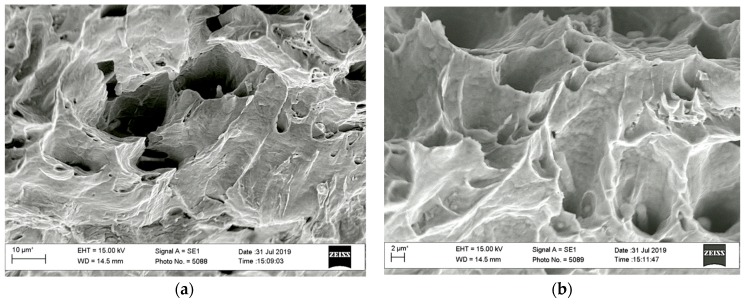
Fractograms of the specimen loaded according to the *K_I_*–*K_III_* scheme (with the *K_III_* type loading up to 45 degrees), at the stage of spontaneous crack growth: (**a**) 10 μm; (**b**) 2 μm.

The characteristic elements of a ductile fracture with a typical dimple relief were observed within the zone of the spontaneous crack growth of tested specimens (Figure 17). It was only noted the significant contribution of shear deformations to the formation of dimples since parabolic ridges delineated them.

It was concluded that regardless of the amplitude of the *K_III_* type loading, the initiation of the fatigue crack growth in the specimens begins from stress concentrators in the middle of their thickness due to *K_I_* type stress effect. Up to a certain depth of crack growth, their propagation rates along this centerline were significantly higher compared to realising ones with approaching to the side surfaces of the specimens. Nevertheless, at the final stage of the starting zones of fatigue crack growth, the local directions of the crack growth on the fracture surfaces deviate away from the centerline (along with the thickness of the specimens) due to *K_III_* type stress effect. In this case, the reorienting of the crack growth direction in the direction of the side surfaces of the specimens was taking place. It means the role of the *K_III_* type loading is to the reorientation of the fatigue crack growth direction.

## 4. Conclusions

The following conclusions from the test results for fatigue crack growth in long operated bridge steel under mixed-mode (I + II, I + III) loading conditions can be drawn:

- Under mixed modes condition, I + II the fatigue lifetime increase with the increase of loading angle θ value. It is caused by the decreasing of the Δ*K_I_*.
- Increase of the angle *α* determining a ratio of the torsional moment to the bending moment causes a decrease of the fatigue life.
- For *α* = 45° and mode I-III, a higher crack growth rate is observed for mode I, which goes into mode III domination. Fatigue crack closure effect evidence is noticeable under mixed-mode I + III configuration, the sliding mode fracture type is observed. However, it is more likely that is caused by damaged fracture surfaces during multiaxial loading.
- The initiation angle is lower than predicted by MTS criteria. It might be caused by the material degradation—microstructure and additional internal stresses, strains (the similar effect was observed by authors in physically prestrained specimens from modern P355NL1 steel [27] and in reported results published in [28]) after 100 years operating time, but this phenomenon should be further investigated.

## Figures and Tables

**Table 1:** Chemical composition of the investigated steel and typical values for puddle iron and old mild steel [18].

Materials	C [%]	Mn [%]	Si [%]	P [%]	S [%]
Investigated steel	0.1	0.52	0.0004	0.028	0.03
Typical values for puddle iron	<0.8	0.4	n/a	<0.6	<0.04
Typical values for old mild steel	<0.15	0.2–0.5	variable	<0.06	<0.15
